# (−)-Guaiol triggers immunogenic cell death and inhibits tumor growth in non-small cell lung cancer

**DOI:** 10.1007/s11010-022-04613-y

**Published:** 2022-11-28

**Authors:** Xiaohui Yang, Junling Yang, Xiaoxia Gu, Yuhua Tao, Hongjuan Ji, Xian Miao, Shuijie Shen, Haiyang Zang

**Affiliations:** 1 Department of Oncology, Nantong Hospital of Traditional Chinese Medicine, Nantong, 226000 China; 2 Research Center of Clinical Medicine, Affiliated Hospital of Nantong University, Nantong, 226001 Jiangsu Province China; 3 Department of Spleen and Stomach, Nantong Hospital of Traditional Chinese Medicine, Nantong, 226000 China

**Keywords:** (−)-Guaiol, ICD, DAMPs, NSCLC, Apoptosis, Autophagy

## Abstract

(−)-Guaiol is a sesquiterpenoid found in many traditional Chinese medicines with potent antitumor activity. However, its therapeutic effect and mechanism in non-small cell lung cancer (NSCLC) have not been fully elucidated. In this study, (−)-Guaiol was found to induce immunogenic cell death (ICD) in NSCLC in vitro. Using (−)-Guaiol in vivo, we found that (−)-Guaiol could suppress tumor growth, increase dendritic cell activation, and enhance T-cell infiltration. Vaccination experiments suggest that cellular immunoprophylaxis after (−)-Guaiol intervention can suppress tumor growth. Previous studies have found that (−)-Guaiol induces apoptosis and autophagy in NSCLC. Apoptosis and autophagy are closely related to ICD. To explore whether autophagy and apoptosis are involved in (−)-Guaiol-induced ICD, we used inhibitors of apoptosis and autophagy. The results showed that the release of damage-associated molecular patterns (DAMPs) was partly reversed after inhibition of apoptosis and autophagy. In conclusion, these results suggested that the (−)-Guaiol triggers immunogenic cell death and inhibits tumor growth in NSCLC.

## Introduction

Lung cancer is one of the most common malignant tumors and a major cause of cancer-related death [1]. Non-small cell lung cancer (NSCLC) is the major subtype of lung cancer, accounting for more than 85% of lung cancer cases [2]. In recent decades, lung cancer treatment based on radiotherapy and cytotoxic chemotherapy has shown therapeutic effects but has resulted in substantial side effects [3]. As research on tumor immune escape mechanisms, increases tumor therapy has gradually shifted to immune regulation [4].

(−)-Guaiol is a sesquiterpene alcohol-containing guaiacane skeleton found in many medicinal plant materials, especially Chinese restorative materials [5]. This molecule has significant bactericidal effects [6–8] and antitumor effects [9, 10] and plays a key antitumor role in some Chinese medicinal materials [11]. This compound can inhibit tumor proliferation and metastasis. Previous studies have indicated that (−)-Guaiol significantly inhibits the growth of NSCLC cells both in vitro and in vivo. In addition, (−)-Guaiol is involved in cell autophagy to regulate the expression of RAD51, leading to double-strand breaks triggering cell apoptosis. These results show that (−)-Guaiol has good antitumor potential [12, 13].

Immunogenic cell death (ICD) is an inflammatory form of cell death that triggers an adaptive immune response. The adaptive antitumor immunity of ICD relies on the release or exposure of damage-associated molecular patterns (DAMPs), including surface calreticulin (CRT), ATP, high-mobility group box 1 (HMGB1), and heat shock proteins (HSP70/90) [14, 15]. DAMPs can activate the recognition of tumor-associated antigens by antigen-presenting cells (APCs), such as dendritic cells (DCs), promote cytotoxic lymphocyte infiltration (CTL), and activate host-specific antitumor immunity. In addition, enhancing the immune surveillance of tumors and eventually leads to long-lasting protective antitumor immunity, which activates immunity to further kill tumor cells [16, 17]. ICD can be triggered via myriad therapeutic agents and interventions (e.g., radiation, viral infection) and may be associated with multiple mechanisms of cell death. Previous reports suggest that the release of DAMPs is closely related to apoptosis and autophagy. CRT exposure is generally believed to mainly occur in the early stage of apoptosis when HSP70 is translocated from the endoplasmic reticulum to the cell surface in the middle of apoptosis [18], and HMGB1 is released from the nucleus. Extracellular metastasis occurs at the late stage of apoptosis [19]. Several studies have shown that ATP exocrine secretion is autophagy-dependent [20]. However, the specific relationship between DAMPs and apoptosis and autophagy remains uncertain. ICD inducers have been the focus of research since the unique killing function of ICD was confirmed [21]. However there are still no effective ICD inducers for NSCLC. [22, 23]. Therefore, exploration and development of ICD inducers in NSCLC is urgently needed.

Since both (−)-Guaiol and ICD are closely related to apoptosis and autophagy, we investigated whether (−)-Guaiol could induce ICD in NSCLC. With in vitro and in vivo studies, we found that (−)-Guaiol could induce DAMP release and inhibit tumor growth. Inhibiting apoptosis and autophagy partly reversed the DAMP release caused by (−)-Guaiol. These results indicate that apoptosis and autophagy might be involved in (−)-Guaiol induced ICD.

## Methods and materials

### Cell culture

The NSCLC cell lines A549 and H1299 and mouse Lewis lung cancer cell lines (LLC) were purchased from the Shanghai Cell Bank of the Chinese Academy of Sciences. These cells were cultured at 37 °C incubators with 5% CO_2_ in DMEM, with 10% FBS and 1% antibiotic. When the cell density reached 70%, 100 uM of (−)-Guaiol (G878838, Macklin) was added to the cells in serum-free medium and the cells were cultured for 24 h or cocultured with 3-methyladenine (3MA) (#S2767, Selleck), or Z-VAD-FMK (#S7023, Selleck) for 24 h.

### Measurement of DAMP production

CRT: Cells were collected with 0.25% trypsin without EDTA, washed twice with cold PBS, and incubated with Alexa Fluor 488 fluorescent anti-calreticulin antibodies in 100 μl of FACS buffer at a 1:100 dilution at 4 °C in darkness for 1 h. The expression of calreticulin (CRT) was analyzed by flow cytometry. ATP: The working fluid was configured according to the instructions of the ATP detection kit (#S0026, Biyuntian). HMGB1: According to the instructions of the high mobility group box-1 protein (HMGB1) ELISA kit (#ARG81351, Arigo), the supernatant of the cells was collected and assessed. HSP70/90: According to the instructions of the HSP70/90 ELISA kit (ADI-EKS-700B, ENZO), the supernatant of the cells to be tested was collected and assessed.

### Confocal imaging

After PBS washes, the antibody was fixed with 4% paraformaldehyde for 20 min, diluted with 5% BSA, added to an appropriate amount of primary antibody working solution and incubated at 4 °C overnight. After PBS washed, the secondary antibody working solution was added dropwise, incubated at 37 °C for 40 min in the dark, and washed with PBS 3 times. DAPI was added to stain the nuclei and incubated at room temperature without light for 30 min. After PBS washes, the tablets were sealed with anti-fluorescence quenching sealing tablets and observed and photographed under a microscope. Anti-calreticulin (#ab92516), Alexa Fluor 488 calreticulin (#ab196158), and β-actin (#ab8226), were purchased from Abcam.

### Animal experiment

C57BL/6 mice (female, 6 weeks old) were purchased from the Animal Experimental Center of Nantong University. LLC cells were subcutaneously inoculated into the right shoulder of the mouse to induce tumor formation. Mice were randomly divided into the control and (−)-Guaiol groups, with 5 mice in each group. One week after injection, the mice in the experimental group were intraperitoneally injected with 8 mg/kg (−)-Guaiol three times a week, and the mice in the control group were intraperitoneally injected with the same amount of NaCl. Tumor growth and body weight were also monitored. The tumor volume was calculated as V (mm3) = (π/6) × A × B2, (A is the longest tumor diameter, B is the shortest tumor diameter). The trial was scheduled to last 28 days from when the tumor was implanted. At the end of the study, the surviving mice were sacrificed under anesthesia. All procedures involving animals and their care are in accordance with the National Institutes of Health Guidelines for the Care and Use of Laboratory Animals. The Ethics Committee of Nantong University approved this study.

### Immunohistochemical (IHC) detection

Paraffin sections were dewaxed and hydrated; then the slides were boiled in sodium citrate antigen repair solution for 10 min and incubated with 3% H_2_O_2_ for another 10 min to inactivate endogenous peroxidase; after that, the slides were blocked with goat serum for 30 min at room temperature, and appropriate primary antibodies were used to incubate the slides overnight at 4 °C. The next day, the secondary antibody was used to incubate the slides for 1 h at room temperature. After that, DAB staining was used to visualize tissue proteins, and hematoxylin was used to counterstain nuclei. CD4(ab183685), CD8(ab209775), CD69(ab202909), CD86(ab234401), CD11c (ab219799) were all purchased from Abcam. MHC II (#68258) was purchased from Cell Signaling Technology.

### Vaccination assay

C57BL/6 mice were randomly divided into 3 groups with 5 mice. LLC cells were treated with or without 100 µM (−)-Guaiol for 24 h and then collected. The cells were repeatedly freeze-thawed on dry ice 3 times. The cells were diluted with the supernatant of the original culture medium, and the cell density was adjusted to 1 × 10^7^ cells/mL. A total of 0.1 ml (1 × 106 cells) was injected subcutaneously into the left scapular region of C57BL/6 mice. The blank group was injected with an equal volume of sodium chloride at the same site. One week later, live LLC cells (1 × 10^5^) were inoculated subcutaneously on the right side of the mouse. Tumor emergence and growth were observed in each group.

### Western-blot assay

Cells were harvested using RIPA lysis buffer mixed with a 1% phosphatase inhibitor complex and 1% protease inhibitor cocktail on ice. After centrifugation, the supernatant protein was collected, and the concentration was determined. The sample was then boiled in SDS for 10 min. Afterward, 40 ug of protein was loaded into an SDS-PAGE gel for electrophoresis. Then, the protein was transferred to NC membranes, blocked with BSA for 1 h, and incubated with primary antibodies at 4 °C overnight. Then, after washes with PBS-T, the membranes were incubated with secondary antibodies for 1 h at 37 °C and washed again, and the bands were visualized using an Odyssey instrument. The gray values of bands were counted by ImageJ, and statistics were performed with SPSS. The antibodies against LC3A/B (#12741) and GAPDH (#5174) were all purchased from Cell Signaling Technology.

### Statistical analysis

GraphPad Prism 8.0 and SPSS 21.0 software were used for plotting and statistical analysis. The quantity and material are expressed as the mean ± standard deviation (SD). One-way ANOVA was used for comparison; *P* < 0.05 indicated a statistically significant difference.

## Results

### *(−)-Guaiol induces DAMP release *in vitro

**Fig. 1:**
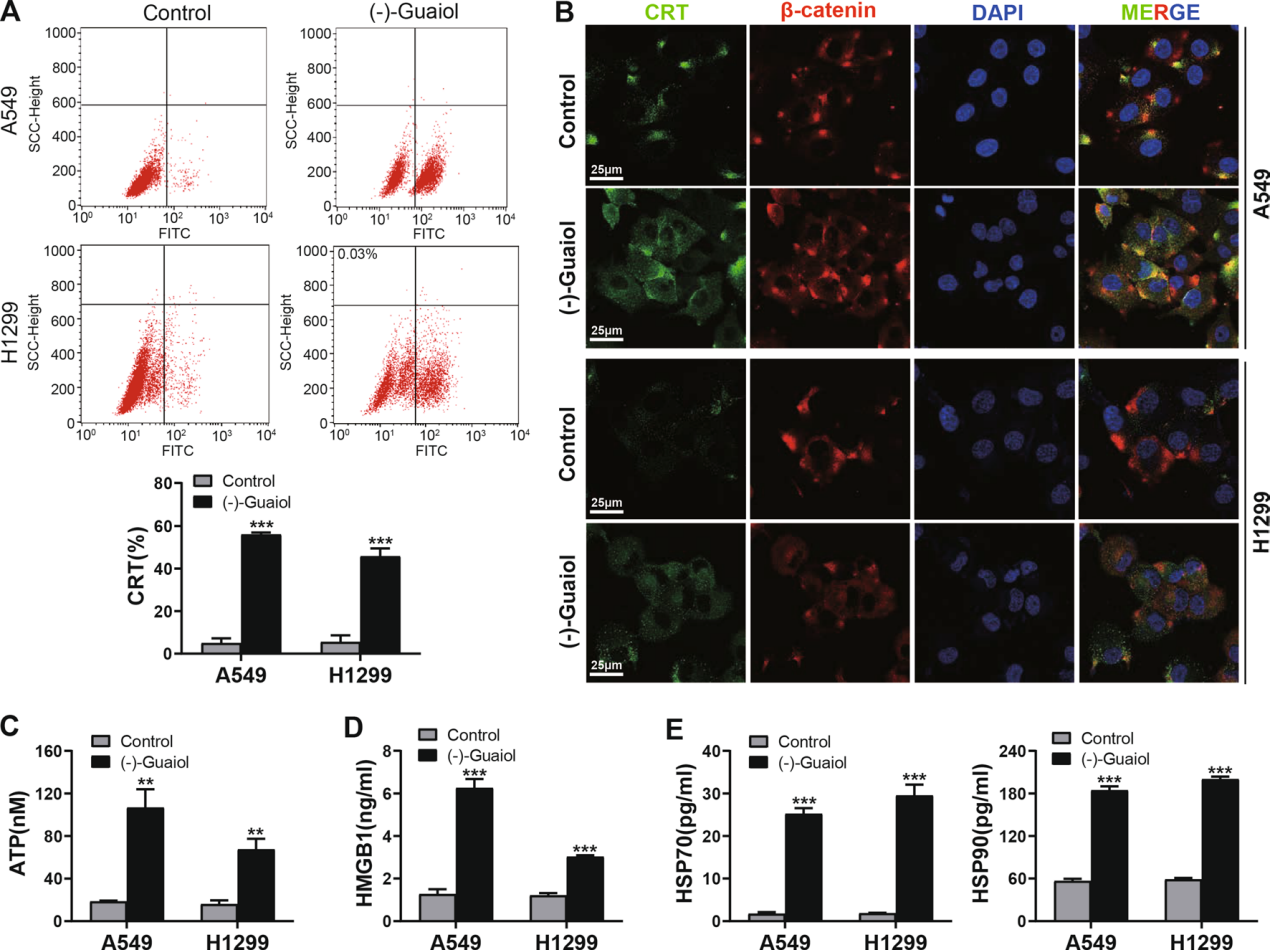
DAMP release after (–)-Guaiol treatment in vitro. **A** The CRT level in three separate experiments detected by flow cytometry. **B** Representative image of CRT location and expression of three confocal images. Green, CRT; Red, β-catenin indicates the cell membrane; Blue, DAPI indicates the nucleus. **C** ATP content in the supernatant detected by an ATP assay kit. **D** Content of HMGB1 in the cell supernatant detected by enzyme-linked immunosorbent assay (ELISA). **E** Content of HSP70/90 in the cell supernatant detected by ELISAs. **p < 0.01, ***p < 0.001 vs. the control group

To investigate whether (−)-Guaiol induces DAMP release in NSCLC, we treated lung cancer A549 and H1299 cells with (−)-Guaiol for 24 h. CRT detection by flow cytometry showed that (−)-Guaiol could increase the positive rate of CRT to at least 30% (Fig. 1A). Immunofluorescence showed that the CRT level on the cell surface, an ‘eat me’ signal that enhances phagocytosis of dying tumor cells [24], was elevated after (−)-Guaiol treatment (Fig. 1B). ATP release, a “find me” signal that acts as a precursor of DC chemical attractant [25], was found to be significantly increased in the cell supernatant (Fig. 1C). HMGB1 can bind to several surface receptors on immune cells and play an essential role as a proinflammatory mediator after it is released from the nucleus to the extracellular cytoplasm [26], and it was significantly increased in the supernatant after (−)-Guaiol treatment (Fig. 1D). Hsp70/90 can form an HSP-peptide complex with tumor-specific antigen peptides, which can present antigens to MHC molecules on the DC surface and induce a tumor-specific immune response [18]. This molecule was significantly increased in the cell supernatant after (−)-Guaiol treatment (Fig. 1E). All these results show that (−)-Guaiol can induce DAMP release in lung cancer cells.

### *(−)-Guaiol suppresses tumor growth and elevates immune infiltration *in vivo

**Fig. 2:**
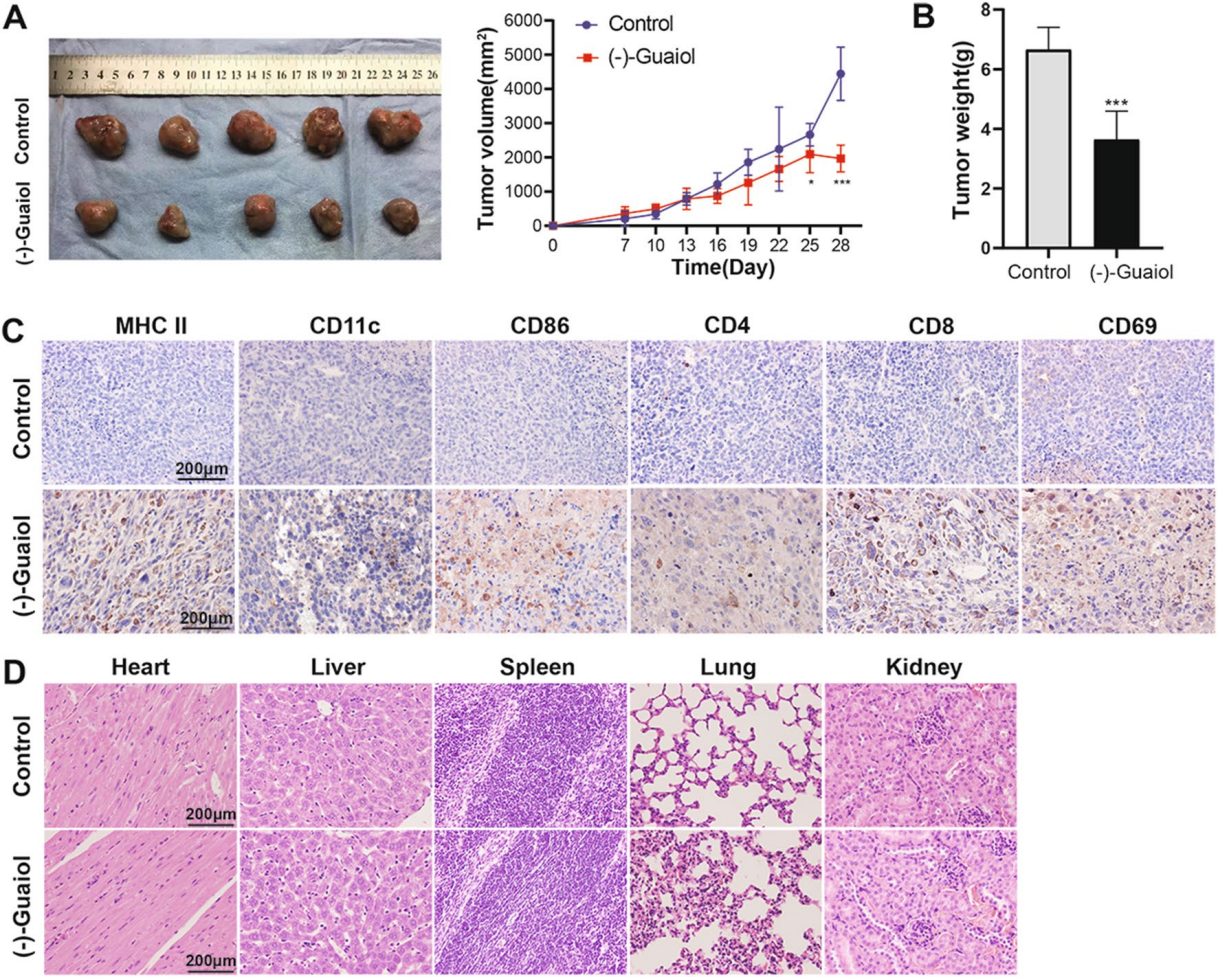
Effect of (–)-Guaiol on tumor growth and immune infiltration in vivo. **A** Tumor volume in the 28 day experiment (*n* = 5). **B** Tumor weight at the end of the experiment (*n* = 5). **C** Representative image of MHC II, CD11c, CD86, CD4+ T, CD8+ T, CD69+ T of three immunohistochemical (IHC) stains. (D) Representative images of three HE stains in the heart, liver, spleen, lung, and kidney. *p < 0.05, ***p < 0.001 vs. the control group

In ICD, dying cells release DAMPs to attract antigen presenting cells such as DCs and induce their maturation, which in turn activates killer T cells and modulates immune-related functions in the immune microenvironment. To evaluate the effect of (−)-Guaiol on antitumor immunity activation in vivo, we established a subcutaneous LLC mouse lung cancer model. The tumor volumes of the mice in both groups were measured during the experiment. The antitumor effect of (−)-Guaiol gradually appeared as the tumor volume at 25 and 28 days was significantly smaller in the (−)-Guaiol treatment group (Fig. 2A). At the end of the experiment, the tumors were removed and weighed. The (−)-Guaiol group had a significantly lighter tumor weight than the control group (Fig. 2B). Then the immune microenvironment was assessed. The expression levels of CD11c, MHC II, and CD86 were increased in the (−)-Guaiol group, and the results suggested that DC infiltration and activation were enhanced. Moreover, CD4+ T cells, CD8+ T cells, and CD69+ T cells were significantly increased, indicating that T-cell infiltration and activation were successfully stimulated after dendritic cell infiltration (Fig. 2C). HE staining of organs showed that (−)-Guaiol was not toxic to the heart, liver, spleen, lung, or kidney (Fig. 2D).

### (−)-Guaiol induces ICD in NSCLC

**Fig. 3:**
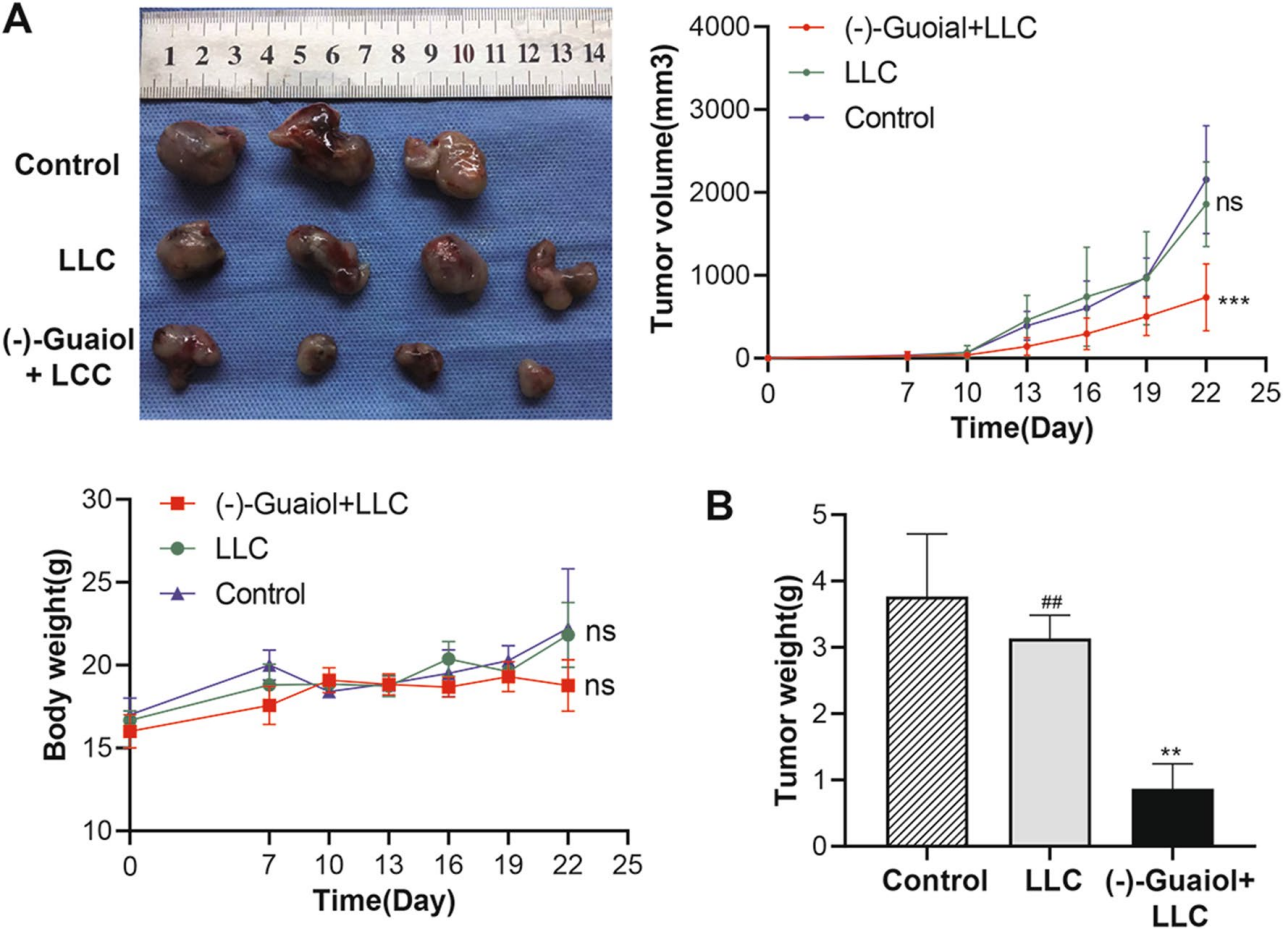
The vaccine effect of (–)-Guaiol in vivo. **A** Tumor volume and body weight during the 22 days of experiment (Control *n* = 3, LLC, n = 4, (–)-Guaiol + LLC, *n* = 4). **B** Tumor weight in the end of the experiment. Ns, not significant vs Control group; **p < 0.01, ***p < 0.001 vs. Control group; ##*p* < 0.01 vs. (–)-Guaiol + LLC Group

Vaccination trials are the gold standard for ICD detection. On Day 22 of tumor implantation, tumor tissue was extracted. As expected, the mice immunized with LLC cells treated with (−)-Guaiol in advance had smaller tumor diameters than the mice vaccinated with sodium chloride and LLC alone (Fig. 3A). Although all mice used in this experiment grew tumors at the end, the tumor weight of the (−)-Guaiol + LLC group was significantly lighter than that of the control and LLC groups, and the results were significant (Fig. 3B). The tumor volume and body weight of the mice in both groups were measured. The results showed that the (−)-Guaiol group had a significantly smaller tumor volume at 22 days, and the body weight of each group showed no difference. All these results indicate that (−)-Guaiol induces ICD in NSCLC.

### Apoptosis inhibitor attenuates the release of DAMPs induced by (−)-Guaiol

**Fig. 4:**
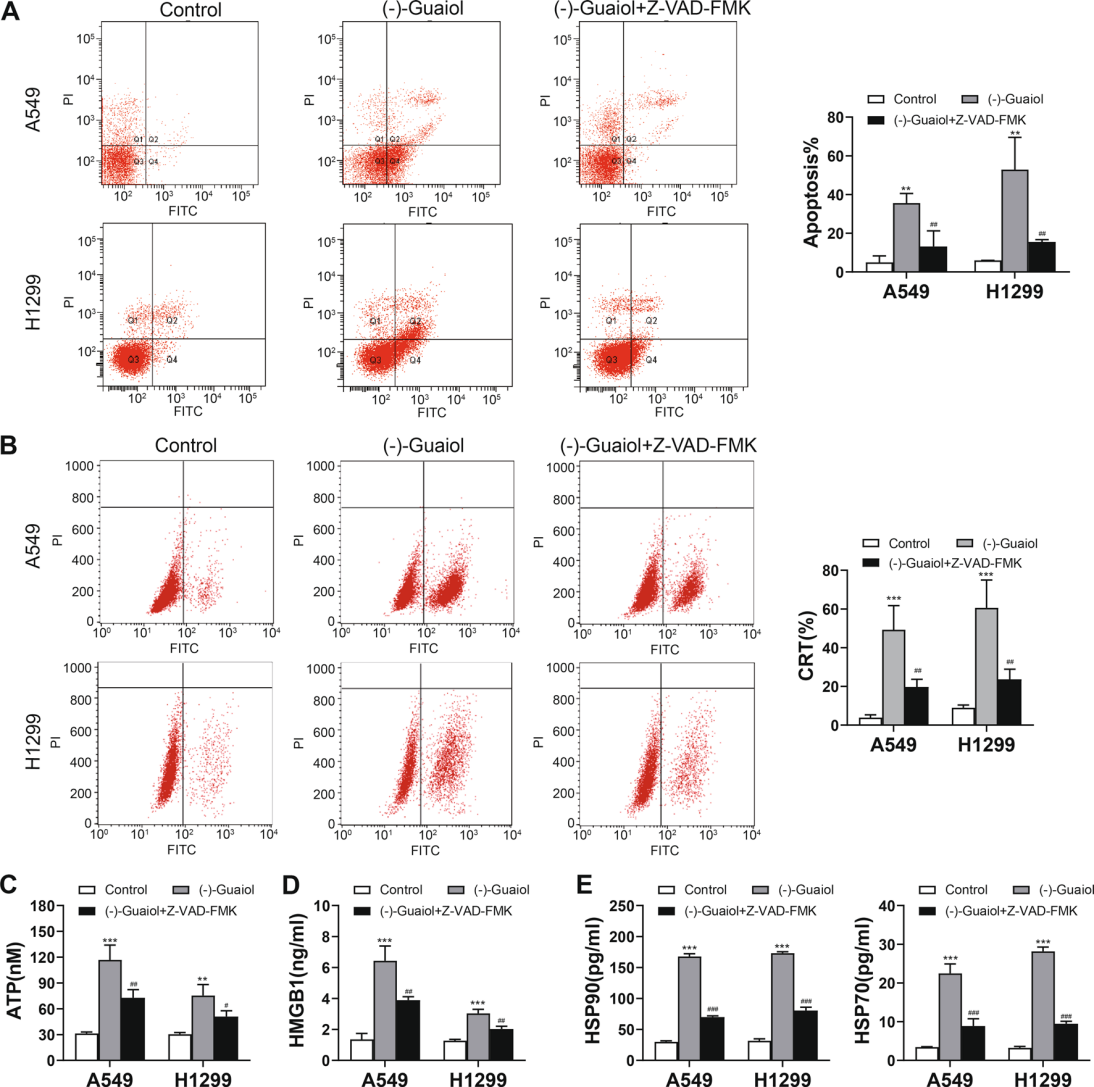
Effect of apoptosis inhibitor on (–)-Guaiol induced DAMP release. **A** Apoptosis detected by flow cytometry. **B** The CRT level detected by flow cytometry. **C** ATP content in the supernatant detected by an ATP assay kit. **D** Content of HMGB1 in the cell supernatant detected by enzyme-linked immunosorbent assays (ELISAs). **E** Content of HSP70/90 in the cell supernatant detected by ELISAs. **p < 0.01, ***p < 0.001 vs. the control group; #*p* < 0.01, ##*p* < 0.01, ###*p* < 0.001 vs. the (–)-Guaiol group

Apoptosis is important for the immunogenicity of cell death and is required for the release of CRT and HMGB1. To determine the role of apoptosis in (−)-Guaiol induced ICD, we used Z-VAD-FMK to inhibit apoptosis and then detected the release of DAMPs. Flow cytometry was conducted to evaluate apoptosis, and the results showed that apoptosis was significantly decreased compared with that in the (−)-Guaiol group after Z-VAD-FMK treatment (Fig. 4A). As expected, CRT (Fig. 4B) and HMGB1 (Fig. 4D) release was significantly decreased after treatment with the inhibitor. Interestingly, the ATP (Fig. 4C) and HSP90/70 (Fig. 4E) were also reduced after treatment with the inhibitor.

### Autophagy inhibitor attenuates the release of DAMPs induced by (−)-Guaiol

**Fig. 5:**
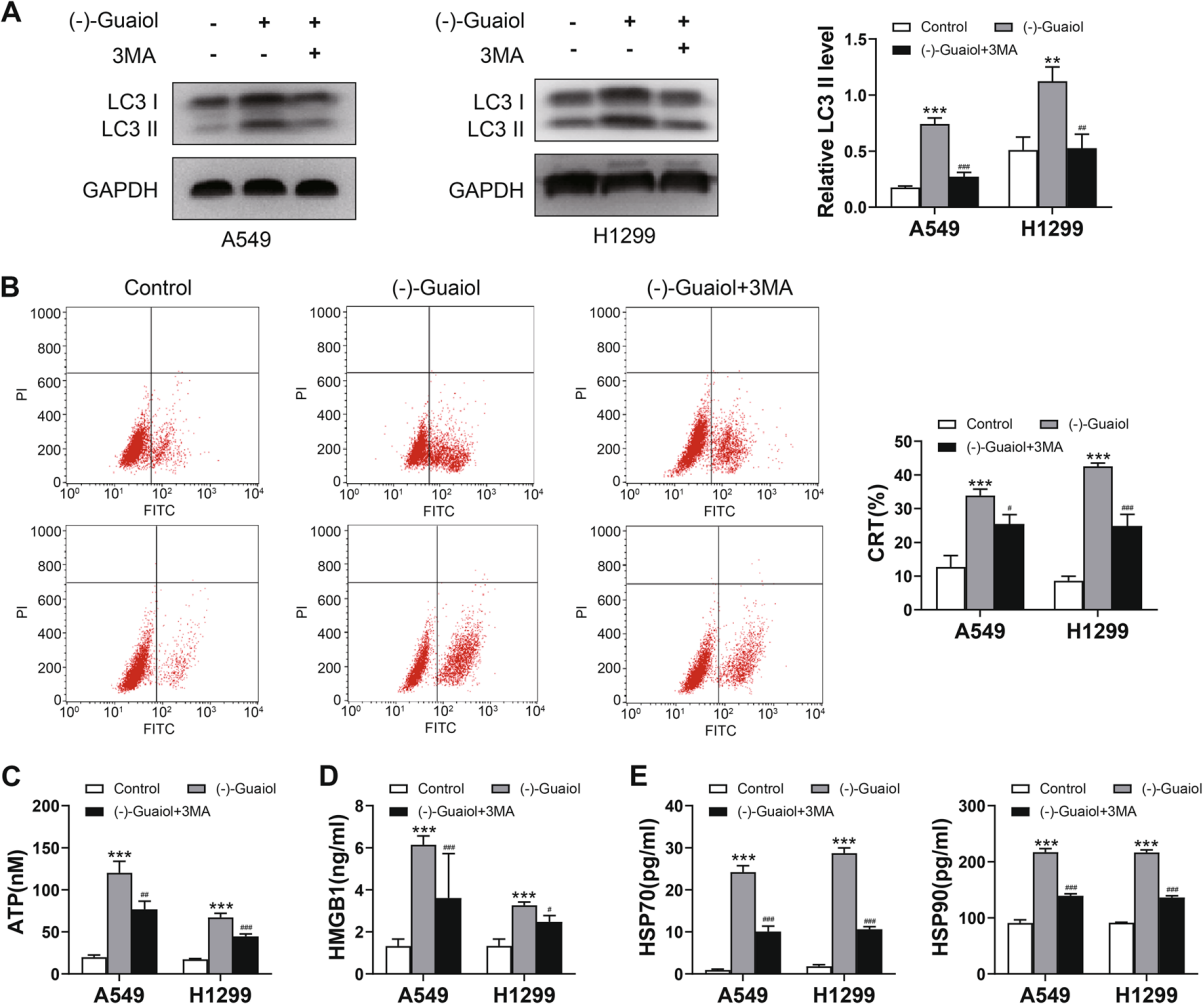
Effect of autophagy inhibitor on (–)-Guaiol induced DAMP release **A** LC3 I and LC3 II detected by WB. **B** The CRT level detected by flow cytometry. **C** ATP content in the supernatant detected by an ATP assay kit. **D** Content of HMGB1 in the cell supernatant detected by enzyme-linked immunosorbent assays (ELISAs). **E** Content of HSP70/90 in the cell supernatant detected by ELISAs. ***p < 0.001 vs. the control group; #*p* < 0.01, ##*p* < 0.01, ###*p* < 0.001 vs. the (–)-Guaiol group

Autophagy is a necessary condition for inducing ICD [27]. To study the role of autophagy in (−)Guaiol induced ICD, we evaluated the autophagy marker LC3. LC3 II was upregulated after (−)-Guaiol treatment but returned to a low level when the cells were treated with the autophagy inhibitor 3MA (Fig. 5A). To test the effect of autophagy on (−)-Guaiol-induced DAPM release, we tested the release of DAMPs. We found that the expression of DAMPs decreased in all tested samples, suggesting that autophagy could not only affect the release of ATP. The autophagy inhibitor 3MA reversed this decrease. This treatment also affected the release of CRT, HMGB1, and HSP70/90 (Fig. 5B–E).

## Discussion

ICD is an attractive therapeutic target that can effectively induce immunity while killing cells and play a dual role in tumor suppression. However, there is no effective ICD inducer in NSCLC treatment. Previous studies have found that (−)-Guaiol is an effective anti-lung cancer drug, showing significant tumor-suppressive effects in vivo and in vitro. We further found that (−)-Guaiol can effectively induce ICD, which is a new ICD-inducing drug worthy of development.

We found that (−)-Guaiol can trigger iconic DAMP release in A549 and H1299 cells. The exocrine secretion of ATP is considered to be a “find me” signal and is an essential condition for ICD [25]. ATP secretion has been reported to be dependent on autophagy. Previous studies confirmed that (−)-Guaiol could effectively induce autophagy. Can it cause ATP extracellular release? To this end, we studied ATP content in the cell supernatant, and as expected, ATP was significantly elevated. CRT on the cell surface can form a complex with ERp57 to provide an "eat me" signal to promote dendritic cell (DC) phagocytosis [24, 28], thereby activating the specific immune response. Our experiment found that (−)-Guaiol could significantly elevate CRT levels on the cell surface. In addition, HSP70/90 can form an HSP-peptide complex with a tumor-specific antigen peptide, which can present tumor antigens to MHC-I molecules on the DC surface and induce a tumor-specific immune response [29]. HMGB1 can bind to multiple surface receptors on disease-free cells and play an essential role as a proinflammatory mediator after being released from the nucleus through the cytoplasm in ICD [30]. All of these molecules are essential parts of DAMPs.

Importantly, ICD enhances antitumor immunity and induces the infiltration and activation of CD8 and other immune cells in the tumor microenvironment [31], in which DCs, as the most functional professional APC in vivo, act as a key regulator of active immune activation [32]. Specific immune responses can be initiated by the binding of the antigen peptide-MHC-II complex to T cells to generate a specific antigen recognition signal (the first signal) and the CD80/CD86-CD28 (the second signal) costimulatory pathway formed by the binding of CD80 on the DC surface and CD86 on the T-cell surface to CD28. Exposure of DAMPs in ICD attracts receptors and ligands on DCs, promotes the transformation of immature DCs into mature phenotypes, enhances the phagocytosis of DCs, stimulates the more lethal specific T-cell response, and increases CTL infiltration in the tumor microenvironment. To activate the adaptive immune response to tumors [14], we detected the expression of CD11c, MHC II, CD86, CD4, CD8, and CD69 in mouse transplanted tumors by IHC and studied the infiltration and activation of DCs and CD4+ T, and CD8+ T cells in the tumor microenvironment after (−)-Guaiol intervention. The results showed that these related indicators are enhanced to varying degrees. CD11c enhancement suggested enhanced expression of DC cells. However, in vivo tumor inhibition experiments proved that (−)-Guaiol had a definite tumor inhibitor effect, and visceral toxicity was not obvious. Notably, the tumor-suppressive effect of this intervention using C57 mice seems to be stronger than that in previous experimental results using nude mice [12], suggesting that the tumor-suppressive effect of (−)-Guaiol may be further strengthened by enhancing and inducing immunity.

Vaccination experiments are the gold standard in ICD detection [33]. Therefore, we conducted a vaccine inoculation experiment and found that LLC cells subcutaneously treated with (−)-Guaiol can significantly slow cancer tumor growth. Thus, we confirmed that (−)-Guaiol could induce ICD through in vivo and in vitro experiments.

Previous study shown that HMGB1 can be transferred to the outside of the cell during apoptosis [34]. And knockdown of key autophagy proteins, such as ATG5, significantly reduces the release of extracellular HMGB1 during necrosis [35]. Other DAMPs, such as ATP, are also regulated by autophagy, ATG5 knockdown inhibits ATP release and prevents a normal immune response [36]. Blocking autophagy attenuates ATP release from chemotherapy-treated cells [25]. We wanted to clarify the relationship between the release of DAMPs induced by (−)-Guaiol and apoptosis and autophagy. For this purpose, we used the apoptosis inhibitor Z-VAD-FMK and the autophagy inhibitor 3MA. The results showed a significant reduction in DAMP release. The results were the same after inhibition of autophagy. However, interestingly, a previous study only found that CRT and HMGB1 release are correlated with apoptosis, and our results suggested that ATP and HSP70/90 were also influenced by apoptosis inhibitors, suggesting that there may be a closer relationship between apoptosis and ICD. Thus, both apoptosis and autophagy are involved in the release of all DAMPs induced by (−)-Guaiol. This finding is consistent with the studies of Karol Prieto [37] and Ye T [38]. In addition, our previous studies have shown that (−)-Guaiol induced autophagy can enhance apoptosis [12], and there may be an interaction between apoptosis and autophagy in the action of this drug. Therefore, it cannot be ruled out that this effect is the result of the interaction between apoptosis and autophagy.

Overall, (−)-Guaiol is a promising new drug for tumor treatment. It has obvious antitumor effects and can enhance immunity and is a promising ICD inducing drug. Our research also found that its ICD inducing effect is correlated with both apoptosis and autophagy, but the exact mechanism underlying this effect requires further investigation.

## Data Availability

Enquiries about data availability should be directed to the authors.
